# Roles and Mechanisms of Hawthorn and Its Extracts on Atherosclerosis: A Review

**DOI:** 10.3389/fphar.2020.00118

**Published:** 2020-02-21

**Authors:** Min Wu, Longtao Liu, Yanwei Xing, Shengjie Yang, Hao Li, Yu Cao

**Affiliations:** ^1^ Guang’anmen Hospital, China Academy of Chinese Medical Sciences, Beijing, China; ^2^ Xiyuan Hospital, China Academy of Chinese Medical Sciences, Beijing, China; ^3^ Institute of Geriatrics, Xiyuan Hospital, China Academy of Chinese Medical Sciences, Beijing, China

**Keywords:** hawthorn, *Crataegus*, atherosclerosis, oxidative stress, inflammation, endothelial dysfunction

## Abstract

Cardiovascular disease (CVD), especially atherosclerosis, is a leading cause of morbidity and mortality globally; it causes a considerable burden on families and caregivers and results in significant financial costs being incurred. Hawthorn has an extensive history of medical use in many countries. In China, the use of hawthorn for the treatment of CVD dates to 659 AD. In addition, according to the theory of traditional Chinese medicine, it acts on tonifying the spleen to promote digestion and activate blood circulation to dissipate blood stasis. This review revealed that the hawthorn extracts possess serum lipid-lowering, anti-oxidative, and cardiovascular protective properties, thus gaining popularity, especially for its anti-atherosclerotic effects. We summarize the four principal mechanisms, including blood lipid-lowering, anti-oxidative, anti-inflammatory, and vascular endothelial protection, thus providing a theoretical basis for further utilization of hawthorn.

## Introduction

Cardiovascular disease (CVD), especially atherosclerosis, is a leading cause of morbidity and mortality worldwide. CVD imposes a considerable burden on families and primary caregivers, along with a high financial cost to society. During past decades in China, ischemic heart disease and stroke are the top two causes of death (Yang et al., 2013). With a rapidly aging population, the absolute number of deaths due to CVD increased by 46% in China, a figure four and three times higher than those in the United States and Western Europe, respectively (Du et al., 2019). Beyond conventional medical treatment, herbal plants have several natural compounds for the prevention and treatment of various diseases. Herbal medicine, such as adjuvants, has also been popular worldwide. It is estimated by the World Health Organization that in the developing countries, nearly four billion people consume herbal medications as a primary source of healthcare (Bodeker and Ong, 2005). Therefore, the use of herbal remedies in complementary and alternative medicines has been widely embraced in many countries (Ekor, 2014).

*Crataegus sp*., commonly known as hawthorn, or hawberry, is a large genus of thorny shrubs and trees belonging to the family *Rosaceae*, comprising approximately 280 species, native to zones with a mild climate in Europe, East Asia, and North America (Hobbs and Foster, 1994). Hawthorn has been used for centuries worldwide as both food and folk medicine. Hawthorn is one of the recognized medicinal plants in European medicine, as Dioscorides primarily described its cardiovascular actions in the first century (Petrovska, 2012). Currently, countries such as China, Germany, and France have officially recorded some species in their pharmacopeias (Chang et al., 2002).

**Figure 1:**
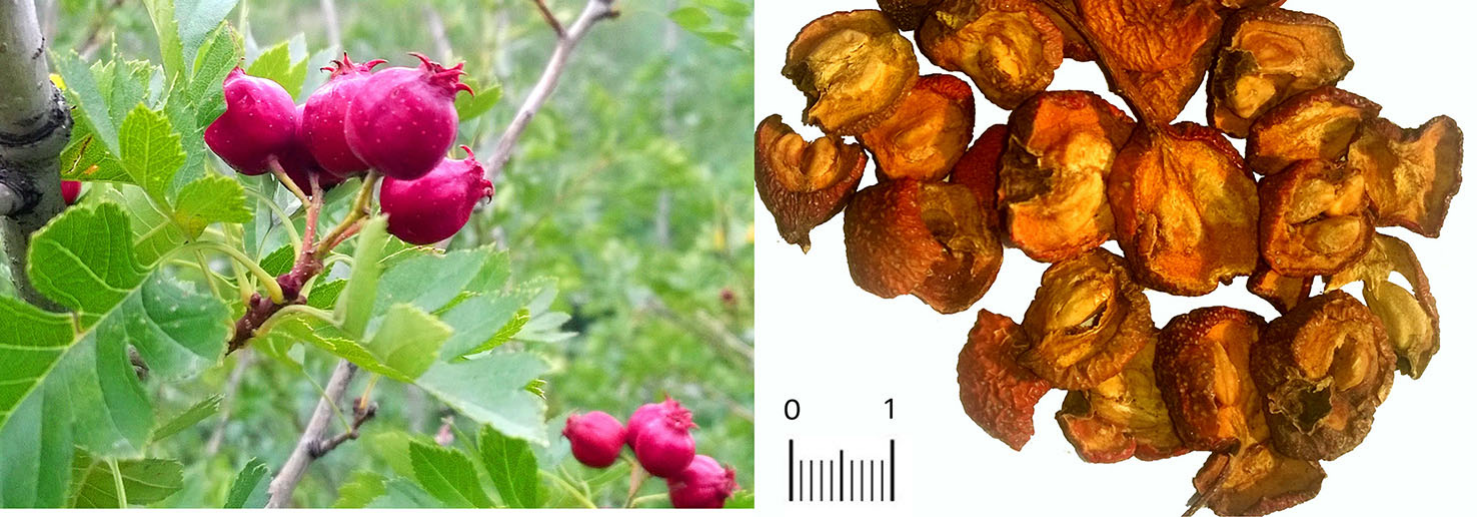
*Crataegus pinnatifida* tree and fruits (left). Traditional Chinese herb *Shanzha* (Fructus *Crataegus*, prepared pieces of *Crataegus pinnatifida* var. *major*) fruit pieces (right).

In China, the bright red berries of hawthorn, also called *Shanzha* (**Figure 1**), have been extensively used to treat various ailments given their medicinal properties. It was mentioned first for “treating dysentery” in *Tang Materia Medica* (*Tang Ben Cao*) dating back to 659 AD, the first known official pharmacopeia in the world. As described in the *Compendium of Materia Medica (Bencao Gangmu*), which is regarded as the most complete and comprehensive herbal monograph, the dried berry of *Crataegus pinnatifida* was described with healing efficacy for thoracalgia, hernia, indigestion, blood stagnation, and hematochezia (Liu et al., 2011). Currently, considerable efforts are underway to identify bioactive components from different parts of the plants and to unveil potential mechanisms of their pharmaceutical actions.

### *Crataegus sp*: Chemical Constituents, Pharmacology, Potential Applications, and Toxicity

Presently, in traditional Chinese medicine (TCM), the fruits of both *C. pinnatifida* Bge. var. *major* N.E.Br and *C. pinnatifida* Bge are the only two medicinal species documented in Chinese pharmacopeia, which are used to promote digestion and improve blood circulation. Other species, such as *Crataegus monogyna* and *C. azarolus*, were also used as focal medicine in other countries. Different parts of the plant, i.e., flowers, leaves, seeds, and berries, have long been recorded for their traditional medical use in either decoction or powder form as folk medicine in many countries, such as Serbia, France, Chile, Turkey, and China, for the treatment of various diseases (e.g., antispasmodic, cardiotonic, diuretic, hypotensive, and anti-atherosclerotic) (Cloud et al., 2019; Dehghani et al., 2019).

**Figure 2:**
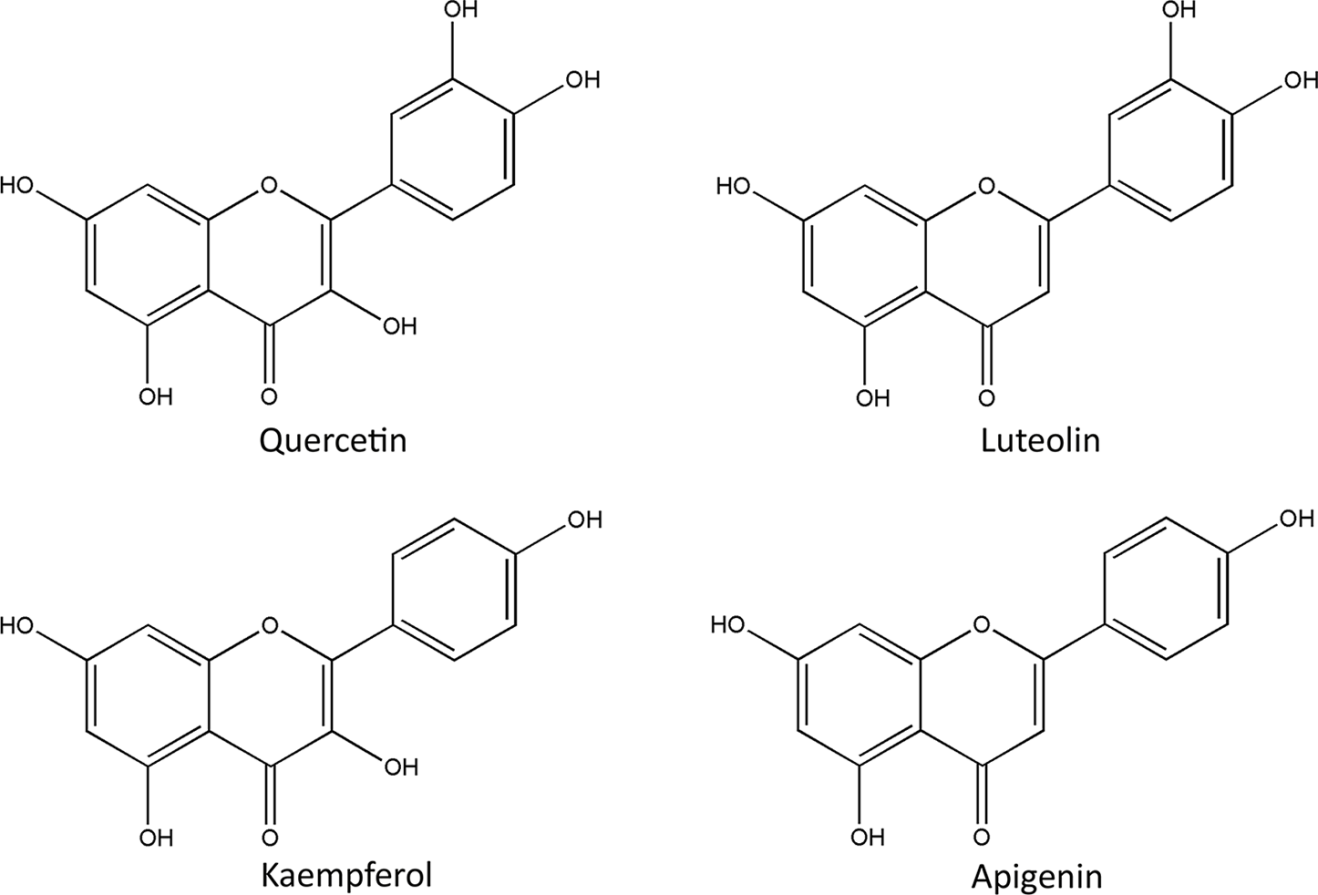
Chemical structures of representative flavonoids in *Crataegus pinnatifida*.

**Figure 3:**
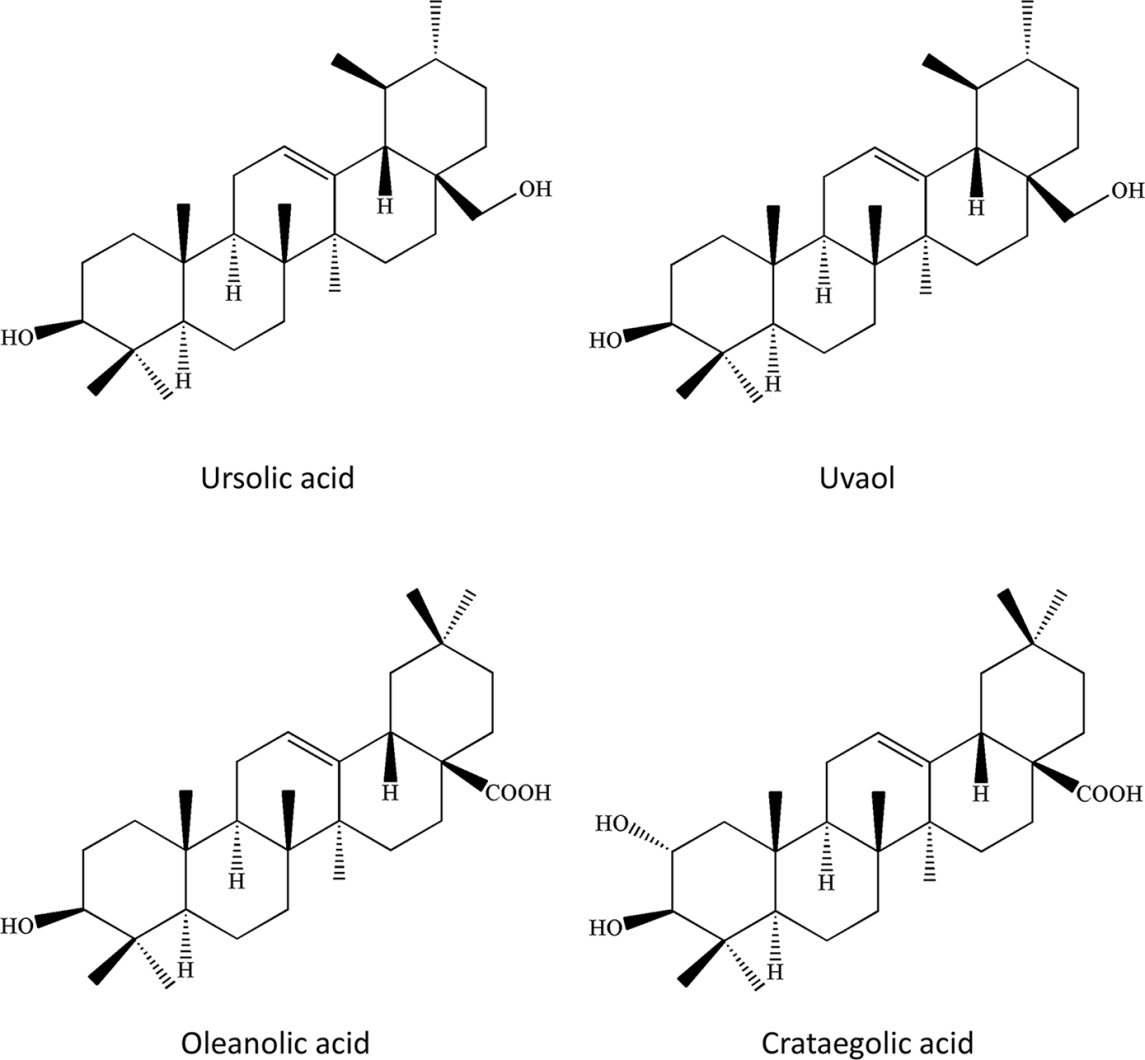
Chemical structures of representative triterpenoids in *Crataegus pinnatifida*.

**Figure 4:**
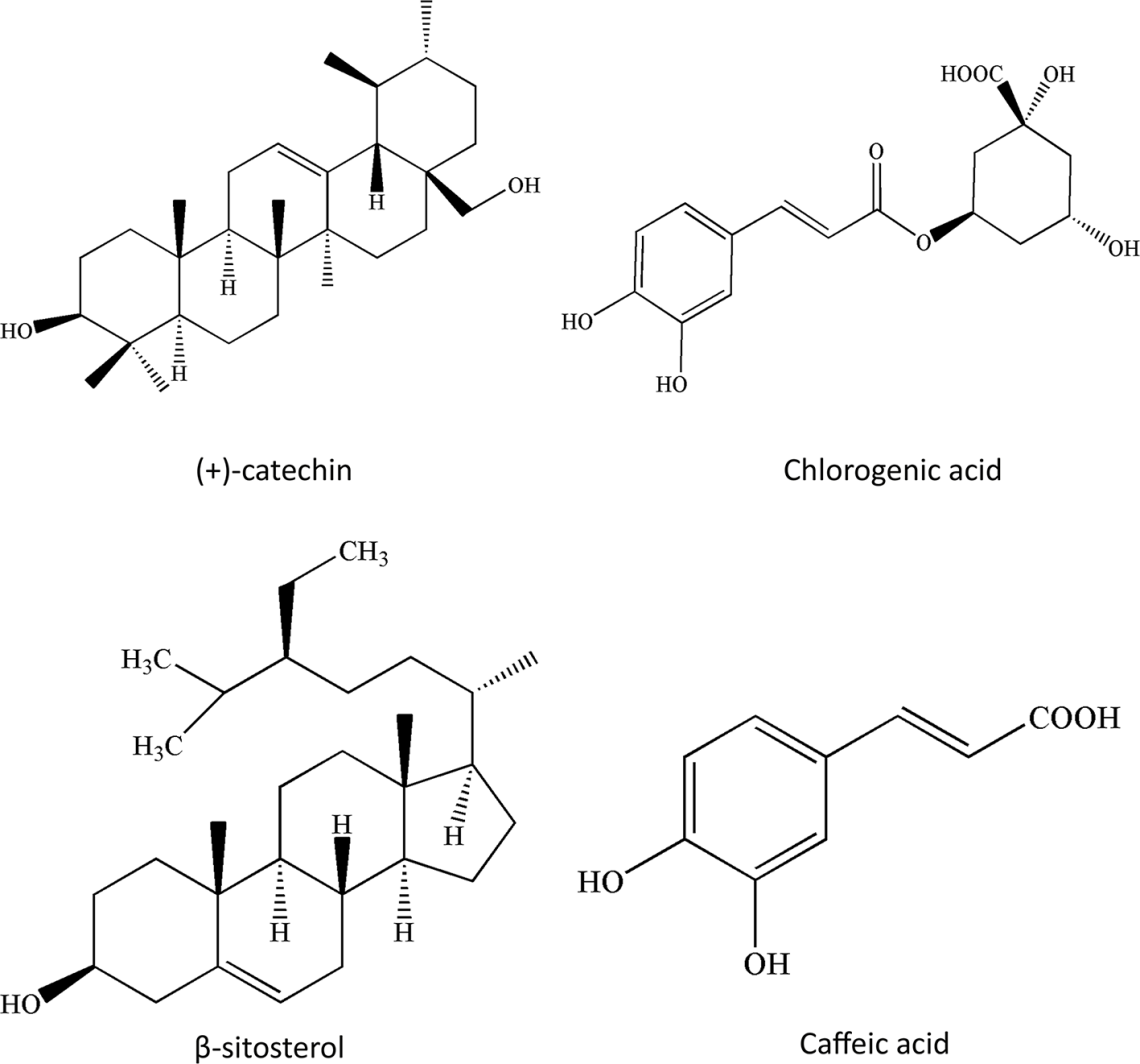
Chemical structures of other compounds in *Crataegus pinnatifida*.

In the past 20 years, greater than 150 chemical compounds, including flavonoids, triterpenoids, oligomeric proanthocyanidins, and organic acids, were separated and characterized in the berries, leaves, and flowers of *C. pinnatifida* (Özcan et al., 2005; Wu et al., 2014) (**Figures 2**–**4**). Moreover, the pectin in fresh hawthorn fruit was reported to be as high as 20.5% (Wang et al., 2007). Pectin oligosaccharides with 2–11 polymers show antioxidant, hypolipidemic, antiglycation, and antibiotic properties (Li et al., 2010; Li et al., 2013a; Li et al., 2014; Zhu et al., 2019). Interestingly, one study showed the contribution of total polyphenolics, rather than the total flavonoids or anthocyanins to the antioxidant capacity of the hawthorn drinks (made from *C. pinnatifida*) (Liu et al., 2016). Besides, heat and microwave exposure could increase the level of anthocyanins, such as cyanidin-3-galactoside (Liu et al., 2016). In other species such as *C. monogyna* and *C. azarolus*, similar types of phenolic compounds were present and categorized into four subclasses: phenolic acids including hydroxycinnamic acids and hydroxybenzoic acid, flavonoids, which are the most abundant components, including flavones and glucosylated flavonols, anthocyanins, such as glycosides of cyanidin, of which cyanidin-3-O-glucoside is the most abundant (Mraihi et al., 2015). Another study identified seven neolignans in the ethanol extract of the *C. pinnatifida* seeds, which elicited antioxidant and anti-inflammatory effects (Peng et al., 2016).

These results show that hawthorn fruits could serve as promising healthcare supplements and also as a potential source of antioxidant and cardiotonic phenolic materials. One study identified the phenolic compounds in the *Crataegus pubescens* fruit, and the most abundant substances were (+)-catechin, (−)-epicatechin, and chlorogenic acid, which could be used as nutraceutical and functional foods (González-Jiménez et al., 2018).

With expanding global interest, modern research validated the presence of multiple biological and pharmacological activities in the extracts of hawthorn fruits, leaves, and flowers, including cardiovascular protective ability, hypolipidemic activity, and anti-oxidative capacity (Pittler et al., 2003; Wang H. et al., 2011; Zhang et al., 2014). WS^®^ 1442 is the most studied compound consisting of 20% oligomeric procyanidins extracted from the leaves and flowers (45% ethanol extract) of *C. monogyna* and *Crataegus laevigata* (Holubarsch et al., 2008). In the United States and European countries, WS^®^ 1442 has been recommended for treating congestive heart failure stages I–III based on the classification of the New York Heart Association (NYHA) (Tauchert, 2002; Pittler et al., 2003).

*Crataegus* sp. has a long history of cardioprotective ability, which is extended for first-line clinical practice. Due to its popularity and efficacy, the extracts of *Crataegus* sp. were assessed in several clinical trials. In addition to its well-known cardiotonic properties, *Crataegus oxyacantha* has also been reported to exert various other pharmacological activities such as anxiolytic, hypotensive, hypolipidemic, antioxidant, hypoglycemic, immunomodulatory, and antimutagenic activities. This article focuses mainly on the anti-atherosclerotic effects of hawthorn and summarizes the mechanisms involved in these effects.

Although no adverse events are reported in its current clinical use, *C. oxyacantha* extracts exhibit genotoxic and mutagenic effects in different cultured cell lines (de Quadros et al., 2017), as well as mild genotoxicity in mice (Yonekubo et al., 2018). Intriguingly, several studies showed a protective effect of *C. microphylla* extracts on a variety of genotoxic insults in lymphoid lineage cells (Hosseinimehr et al., 2006; Hosseinimehr et al., 2008; Hosseinimehr et al., 2009; Hosseinimehr et al., 2011). These results suggest caution regarding prolonged or high-dose use.

### Hypolipidemic Activity

CVDs have been the leading cause of mortality and morbidity globally for decades. The primary pathogenesis of CVDs is atherosclerosis, which could lead to dramatic clinical events, such as unstable angina or myocardial infarction (Reiner et al., 2011). The underlying pathophysiological mechanisms of atherosclerosis are oxidative stress damage, lipid deposition, inflammatory responses, and vascular endothelial dysfunction (Libby et al., 2002; Weber and Noels, 2011). Multiple risk factors associated with the development of atherosclerotic plaque are diabetes mellitus, dyslipidemia, hypertension, obesity, and cigarette smoking (Folsom et al., 1997; Hackam and Anand, 2003; Mannarino and Pirro, 2008). Among the risk factors identified in epidemiological studies, only apolipoprotein (apo)-B containing lipoproteins, including low-density lipoproteins (LDLs) and very-low-density lipoproteins (VLDLs), cause atherosclerosis in both humans and experimental animals (Skålén et al., 2002).

Once the LDL traverses to the sub-endothelium, it binds to the chondroitin sulfate (CS) and dermatan sulfate (DS) site of the matrix proteoglycans, further leading to entrapment and phagocytosis by macrophages and other cells, eventually forming the foam cells and lipid core to initiate the local inflammatory response (Little et al., 2007). Lipid retention is an essential and critical initial step in the atherosclerotic cascade, and without this event, atherosclerosis could not be induced in animal models (Olofsson and Boren, 2005).

A 6-month clinical observation of 64 patients with carotid artery atherosclerosis showed that the ingestion of hawthorn extract at a dose of 5.0 mg/kg reduces the level of serum lipid and promotes plaque stability (Liu et al., 2014). *C. pinnatifida* extracts that primarily contain flavonoids showed promising hypolipidemic activity in different experimental animals. Flavonoid extracts from the leaves of *C. pinnatifida* considerably decreased level of the serum lipids, such as total cholesterol (TC) and triglyceride (TG) in rats, mice, and rabbits (Zhang et al., 2002b; Luo et al., 2009; Zhang et al., 2013). The reduction in the level of serum total cholesterol and TG is a complex process involving multiple steps in cholesterol metabolism. First, it inhibits cholesterol absorption by downregulating the expression and activity of intestinal acyl-CoA cholesterol acyltransferase (ACAT). Intestinal ACAT is a critical regulator involved in cholesterol absorption by esterification of cholesterol before absorption (Zhang et al., 2002a). Second, the total flavonoids attenuate the expression of two essential liver enzymes involved in lipid biosynthesis, hydroxy methylglutaryl coenzyme A reductase (HMG-CoA) and cholesterol-7-alpha-hydroxylase (CYP7α) (Zhang et al., 2002a; Kwok et al., 2013). Moreover, the total flavonoids of *C. pinnatifida* inhibit the mature adipocyte secretion of the leptin and plasminogen activator inhibitor (PAI)-1 (Liu et al., 2009c) and decrease adipogenesis-related gene expression, including sterol regulatory element-binding proteins-1c (SREBP), fatty acid synthase (FAS), triacylglycerol hydrolase (TGH), and hormone-sensitive TG lipase (HSL) (Xie et al., 2009). A more recent study showed consistent results using extracts derived from the leaves of *C. pinnatifida* [Hawthorn leaf flavonoids (HLF)]. HLF decreased serum lipid levels, including total TGs, cholesterols, and lipoproteins, such as VLDLs and LDLs in Apoe^-/-^ mice due to the involvement of the hepatic lipid metabolism-related genes, including peroxisome proliferator-activated receptor (PPAR)α, SREBP-1c, CPT-1, and HMG-CoAR reductase (Dong et al., 2017). *In vitro* data showed HLF inhibited the formation of foam cells by promoting cellular cholesterol efflux *via* the upregulation of the ATP-binding cassette transporter A1 (ABCA1), PPARγ, and downregulation of CD36, thus preventing the progression of atherosclerotic lesions (Liu et al., 2009a). Another line of research showed that pectin oligosaccharides and pectin hydrolysates fractionated from *C. pinnatifida* could restore unbalanced cholesterol metabolism and serum lipid overload in mice or hamsters that are fed a high-fat diet (HFD) (Li et al., 2010; Li et al., 2014; Zhu et al., 2015). This is specifically by the involvement of hepatic enzymes, such as glycerol 3-phosphate acyltransferase (GPAT), SREBP-2, phosphatidate phosphohydrolase (PAP), cholesterol-7α-hydroxylase (Zhu et al., 2013; Li et al., 2014), and hepatic fatty acid oxidation-related enzyme activities of acyl-CoA oxidase, 3-ketoacyl-CoA thiolase, 2,4-dienoyl-CoA reductase, and carnitine palmitoyltransferase I (Li et al., 2013b), and these results are consistent with earlier reported data. For example, pectin penta-oligogalacturonide was shown to increase the expression of CYP7A1, bile salt export pump, but not the sodium-taurocholate co-transporting polypeptide (Zhu et al., 2017). Similarly, pectin also reportedly downregulated the abnormal activity of high fat-induced SREBF-1c, suppressing the protein expression levels, pyruvate kinase, acetyl-CoA carboxylase, and fatty acid synthase enzyme activities (Li et al., 2017). Altogether, these data showed that pectin and its derivatives may be promising candidates for the treatment of hyperlipidemia and atherosclerosis.

Most of the research was conducted with the European and East Asian species. There exists little knowledge on plants native to other parts of the world. One study on the extracts from berries and leaves of *Crataegus chrysocarpa*, a species native to North America, showed that a reduced serum fasting LDL-C improved heart function by elevating the nitric oxide (NO) levels (Diane et al., 2016).

### Anti-Oxidative Activity

Although elevated serum lipid levels are essential for atherosclerosis progression, LDLs in their native state cannot trigger the lipid retention cascade before oxidization by free radicals. Therefore, oxidative stress is crucial to illustrate atherogenic mechanisms. Oxidative stress is a state of unbalanced tissue oxidation due to the disturbed equilibrium between pro-oxidant and antioxidant molecules, an essential step in the pathophysiological development of atherosclerosis (Stocker and Keaney, 2004). Analysis of the atherosclerotic lesion compositions showed the oxidative products of protein and lipid such as F2α-isoprostanes, chlorinated lipid hydroperoxides, short-chain aldehydes, nitrated amino acids, oxidized phospholipids, and oxysterols as the major constituents (Heinecke, 1998). Recent studies, both *in vivo* and *in vitro*, showed that the extracts of *C. pinnatifida* eliminate free radicals to attenuate LDL oxidation (Wang T. et al., 2011; Cheng et al., 2013; Bedreag et al., 2014). In contrast to native LDLs, only the modified or oxidized LDLs can drive the development of atherosclerosis. It has been demonstrated that extracts from *C. pinnatifida* exert potent scavenging properties against DPPH, hydroxyl radicals, and copper-II and peroxyl radical-induced LDL cholesterol oxidation (Zhang et al., 2001; Liu et al., 2009b), as well as hydrogen peroxide and superoxide species (Bahorun et al., 1996), which is partially due to interactions with antioxidant enzymes, such as superoxide dismutase (SOD) and glutathione peroxidase (GSH-px) (Wang W. et al., 2011; Zhang et al., 2014). The pectin oligosaccharides of the *C. pinnatifida* fruit also reduced the aberrant oxidative state in a mouse model of experimental hyperlipidemia, as indicated by reduced serum synthesis and accumulation of malondialdehyde and increased SOD levels (Li et al., 2010). Metabolomic results showed that seedless hawthorn fruit extracts increased the levels of 11 different metabolites related to oxidative responses, as well as the concentration of NO and the activity of NO synthase, which are known factors that prevent against oxidative impairments (Zheng et al., 2019). The mechanism may involve the nuclear factor erythroid 2–related factor (Nrf2)/heme oxygenase-1 (HO-1) signaling pathway, major sentinels, and effectors in response to the oxidative status (Yoo et al., 2016). Similarly, another study provided evidence that the total flavones of hawthorn mitigate the endothelial cell impairment after coronary bypass graft operation by reducing the oxidative stress (Zhu et al., 2018). In addition, extracts of *C. oxyacantha* attenuate the ischemia–reperfusion insults, and the potential mechanism might be attributed to a reduction in the oxidative stress level, which increases after ischemia–reperfusion injury (Ranjbar et al., 2018). Furthermore, vitexin (an extract from hawthorn leaves) suppressed doxorubicin (DOX)-induced oxidative stress, inflammation, apoptosis, and myocardial damage through a mechanism involving increased cellular expression of p-FoxO3a (Sun et al., 2016). Thus, the Akt/FoxO3a signaling pathway may be a novel target for the development of drugs to reduce DOX-induced cardiotoxicity.

### Endothelium Protection

Endothelium dysfunction is a well-known independent risk factor for coronary heart disease and is closely associated with its clinical outcome (Daiber et al., 2017). Endothelium dysfunction occurs in the early stages of atherogenesis, characterized by a reduction of NO-mediated vasodilator responses and increased vasoconstriction due to excess endothelin (ET)-1 synthesis, resulting in enhanced vascular permeability. Such alterations lead to increased cell permeability, the release of pro-inflammatory factors, such as adhesion molecules and cytokines leukocyte adherence, platelet hyper-activation, enhanced low-density lipoprotein oxidation, as well as vascular smooth muscle cell proliferation and migration (Corte et al., 2016; Costantino et al., 2016; Jamwal and Sharma, 2018).

Hawthorn extracts resulted in decreased ET-1 and elevated NO levels in both human and animal experiments (Asher et al., 2012; Zhang et al., 2013). Endothelial NO synthase (eNOS) is a critical regulator of NO synthesis. The translocation from the cell membrane results in increased phosphorylation of serine residue 1177 or decreased phosphorylation of threonine residue 495, which are pathways for activating eNOS. Hawthorn extracts could induce vasorelaxation by increasing the phosphorylation of serine residue 1177 (Brixius et al., 2006). Hawthorn extract WS^®^ 1442 increases cytosolic [Ca^2+^] i by suppressing sarcoplasmic/endoplasmic reticulum Ca^2+^ ATPase (SERCA) and activating the inositol 1,4,5-trisphosphate (IP3) pathway, but without affecting the barrier function or endothelial cell contraction. More importantly, it does not induce store-operated calcium entry (Willer et al., 2012).

In other pathological conditions attributed to atherogenesis, such as diabetes, aging, and hypertension, hawthorn extracts have shown a promising effect in maintaining the integrity and normal endothelial function both *in vitro* and *in vivo* (Xiyun and Yaofa, 2002; Bubik et al., 2012; Idris-Khodja et al., 2012; Peters et al., 2012; Topal et al., 2013).

### Anti-Inflammatory Activity

Over the past decades, lipid retention and oxidation are regarded as the *sine qua non* of the atherosclerosis (Moriya, 2019), and how and where the lipid oxidation occurs have not been identified. Chemical analysis of the modified lipids and proteins, using human atheroma samples, does not necessarily correspond to the transition metal-mediated oxidized lipoproteins *in vitro*; also, randomized clinical trials failed to validate the effectiveness of antioxidant therapy (Pascual-Teresa et al., 2010; Myung et al., 2013).

Recent atherosclerosis studies focused on inflammation, thus providing new insight into the mechanisms of this disease. Inflammation participates at all stages of atherosclerosis development. Clinical research has identified the critical role of inflammation in the development and progression of coronary artery disease. Immune cells are present in early atherosclerotic lesions, and their effector chemicals drive the development of the lesions. The hyper-activated inflammatory response can lead to acute coronary syndromes (Gimbrone and García-Cardeña, 2016; Ferrucci and Fabbri, 2018; Ridker, 2019). Our previous study showed that hawthorn extracts alleviated atherosclerosis by inhibiting inflammation and apoptosis-related factors (Wang et al., 2019).

An *in vitro* study on mouse macrophage cell line showed that an aqueous extract of hawthorn fruit suppressed expression of inflammatory cytokines, such as interleukin (IL)-1β, tumor necrosis factor (TNF)-α, and IL-6 (Kim et al., 2011), which may be regulated by the nuclear factor (NF)-κB, a well-known mediator for its role in pro- and anti-inflammatory regulation and for controlling the expression of inflammatory genes including adhesion molecules, cytokines, and chemokines. Data from *Crataegus aronia* extracts showed inhibition of the nuclear accumulation of NF-κB and NLRP-3 protein levels and caspase-1 in HFD-induced aortic vascular inflammation in rats, thus suggesting inhibition of the NLRP3 inflammasome-mediated pathway (Shatoor and Al Humayed, 2019). The monocyte/macrophage is a major immunocyte involved in atherosclerosis, which scavenges modified LDLs and accelerates the local inflammatory response (Kao et al., 2005). One study reported that water extracts of *C. pinnatifida* inhibited NO production and inflammatory gene expression, including TNF-α, COX-2, IL-1β, and IL-6 in lipopolysaccharide (LPS)-stimulated RAW 264.7 cells (Li and Wang, 2011). In addition, ethanol extracts of *C. pinnatifid*a seeds elicit a potent NO and TNF-α inhibitory effect, hence regarded as a promising and reliable source of antioxidants and inhibitors of inflammation (Peng et al., 2016). Clinical evidence showed that when metformin was combined with hawthorn, the level of body mass index (BMI), HbA1c, FPG, 2hPG, TG, and hs-CRP remarkably reduced in patients with prediabetes complicated by nonalcoholic fatty liver disease (Gao et al., 2019). Altogether, these studies demonstrate that hawthorn extracts may be a promising drug for the treatment and prevention of atherosclerosis.

In addition to the activities mentioned above, the newly identified norditerpenoids from the leaves of *C. pinnatifid*a exert antithrombotic activities both *in vitro* and *in vivo* (Gao et al., 2017). The norditerpenoids prevent adenosine diphosphate-induced platelet accumulation, mediated in response to the purinergic P2Y receptor, and delay thrombocyte aggregation induced by FeCl_3_ in the caudal vessels of zebrafish (Gao et al., 2017). Antiplatelet and antithrombotic treatment is a critical approach for the treatment of acute coronary artery syndrome and secondary prevention of coronary heart disease, as recommended by the global guidance for CVD (Vandvik et al., 2012; Stewart et al., 2017).

However, hawthorn ingestion could lead to higher bleeding risk, and a clinical study of patients who underwent cardiac surgery reported that hawthorn extract consumption may increase the postoperative bleeding rate, the amount of chest tube output after cardiac surgery, and most importantly, the overall mortality (Rababa’h et al., 2016). Since the use of antiplatelet and antithrombotic medications are quite common in the treatment of CVDs, such as atherosclerosis, arrhythmia, and post-percutaneous coronary intervention, patients with such diseases should exercise caution while using hawthorn as an adjuvant medication. Further high-grade evidence is also required to assess the safety of hawthorn products in the treatment of CVDs.

**Figure 5:**
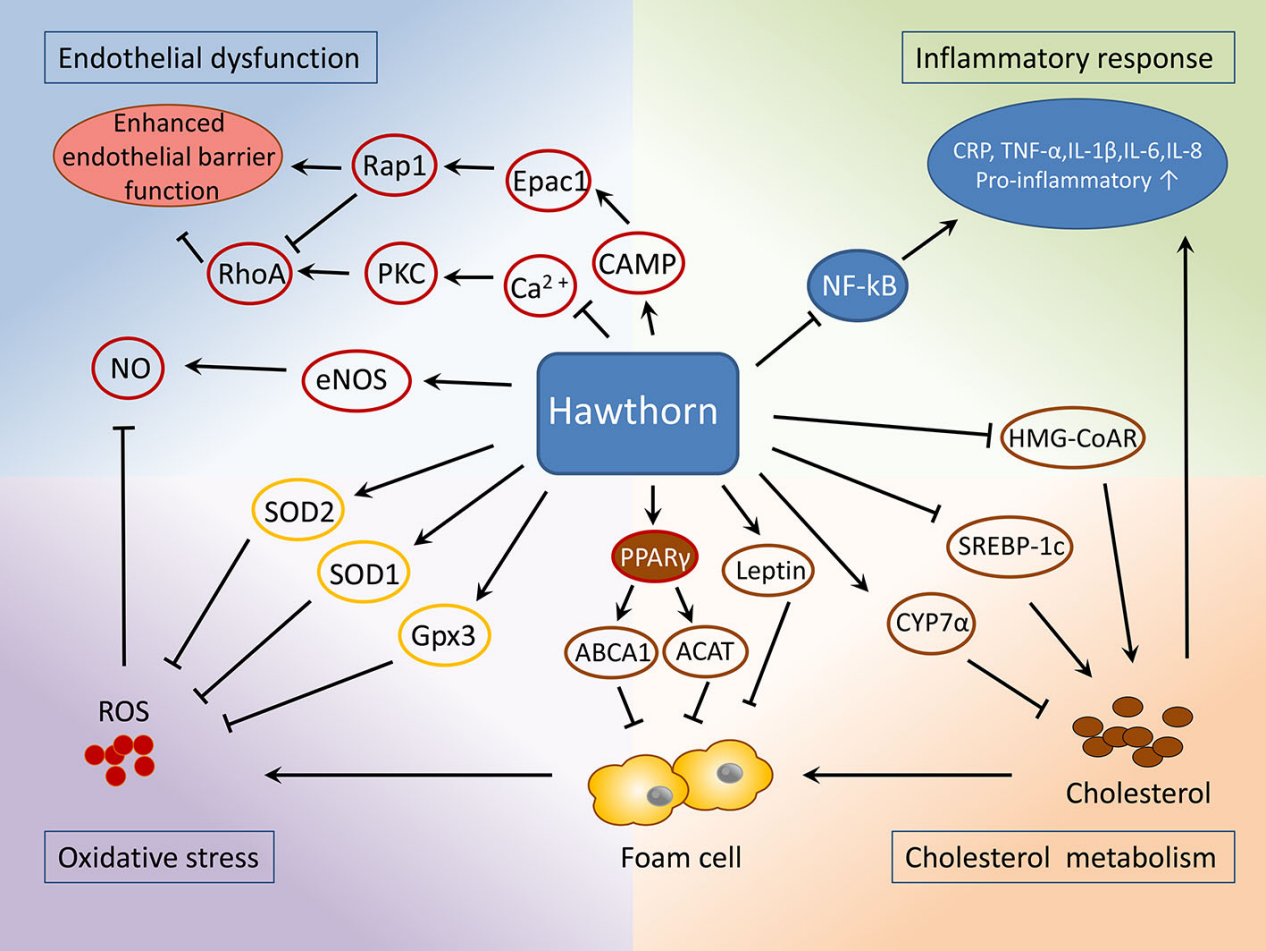
Mechanisms of the protective role of hawthorn in the treatment of atherosclerosis.

Apart from these actions, one more recent study showed that a 16-week oral administration of *C. pinnatifida* leaf extracts suppressed the overall apoptotic level in the aorta, as indicated by the alternations of the expressions of Bcl-2 (B-cell lymphoma 2) and BAX (BCL2-associated X protein) (Wang et al., 2019). Besides, hawthorn extracts were reported to process an antiapoptotic effect in ischemic myocardial damage (Jayachandran et al., 2010; Ranjbar et al., 2018), a common result due to atherosclerotic lesions invading coronary arteries. This may attribute to the activation of Akt and HIF-1 (hypoxia-inducible factors) signaling pathways (Jayachandran et al., 2010). Myocardial apoptosis is known as a key pathological feature under ischemic conditions. Strategies against apoptotic cascades have been proposed for the treatment of ischemic heart disease (Perricone and Vander Heide, 2014). To this end, hawthorn extracts may be promising complementation for the treatment of cardiac ischemic injuries as well as post myocardial infarction rehabilitation. (The cardiovascular effects are shown in **Table 1** and **Figure 5**.)

**Table 1:** Major effects and targets of *Crataegus* in atherosclerosis.

Effects	Species	Materials	Subjects	Targets	Reference
Lipid-lowering effect	*C. pinnatifida*	Freeze-dried fruit powder	ApoE^-/-^ mice	ACAT	Zhang et al., 2014
				SREBP-1c	
				HMG-CoAR, CYP7α	
				FAS	
				PPARα, CPT-1	
		Fruit powder	New Zealand rabbits	HMG-CoAR, CYP7α ACAT	Zhang et al., 2002b
		Ethanol extracts	Syrian golden hamsters	HMG-CoAR	Zhang et al., 2002a
				ACAT
		Total flavonoids	3T3-L1 cells	Leptin	Liu et al., 2009c
	*C. pinnatifid*a var. *major*	50% ethanol extracts of leaf	Macrophage of ApoE^-/-^ mice	PPARγ, ABCA1, CD36	Liu et al., 2009a
Antioxidant effect	*C. pinnatifida*	Freeze-dried fruit powder	ApoE^-/-^ mice	SOD1, SOD2, Gpx3	Zhang et al., 2014
	*C. pinnatifida* var. *major*	Aqueous extracts of fruits	Wistar rats	SOD, MDA	Wang W. et al., 2011
Endothelial protection	*C. pinnatifida* var. *major*	Aqueous extracts	Wistar rats	NO, ET	Zhang et al., 2013
				6-keto-PGF1α, TXB2	
	*C. monogyna* and *C. laevigata*	WS^®^ 1442	Rat aorta and human internal mammary artery	Serine 1177 residual of eNOS	Brixius et al., 2006
			HUVECs, C57 mice	cAMP/Epac1/Rap1 pathway Ca^2+^/PKC/RhoA signaling pathway	Bubik et al., 2012
			HUVECs	IP3 Sarcoplasmic/endoplasmic reticulum Ca^2+^ ATPase	Willer et al., 2012
Anti-inflammatory effect	*C. pinnatifida* var. *major*	50% ethanol extracts of leaf	Carotid artery atherosclerosis patients	CRP	Liu et al., 2014
		Aqueous extracts of fruits	Wistar rats	IL-18	Zhang et al., 2013
				CRP, IL-1β, IL-8	
	*C. oxyacantha*	Hawthorn vinegar	Cardiovascular risk patients	CRP	Kadas et al., 2014
	*C. pinnatifida* var. *typica*	Methanol extracts	RAW 264.7 cell	TNF-α, IL-1β, IL-6	Tauchert, 2002
	*C. pinnatifid*a var. *major*	50% ethanol extracts of leaf	ApoE^-/-^ mice	CRP, NF-κB	Zheng and Wang, 2010
Anti-apoptotic effect	*C. pinnatifida* var. *major*	50% ethanol extracts	ApoE^-/-^ mice	BAX, Bcl-2	Wang et al., 2019
	*C. oxyacantha*	Heartcare™ tablets	Wistar rats	Akt and HIF-1 signaling pathways	Jayachandran et al., 2010

6-keto-PGF1α, 6-keto-prostaglandin F1α; ABCA1, ATP-binding cassette transporter 1; ACAT, acyl-CoA cholesterol acyltransferase; Bcl-2, B-cell lymphoma 2; BAX, BCL2-associated X protein; CPT-1, carnitine palmitoyltransterase-1; CRP, C-reactive protein; ET, endothelin; Gpx3, glutathione peroxidase 3; HIF-1, hypoxia-inducible factor 1; HUVECs, human umbilical vein endothelial cells; MDA, malondialdehyde; NF-κB, nuclear factor κB; NO, nitric oxide; PPAR, peroxisome proliferator-activated receptor; SOD, superoxide dismutase; TNF-α, tumor necrosis factor α; TXB2, thromboxane B2.

## Conclusions

Medicinal plants possessing considerable quantities of therapeutic natural products are considered a promising source of medicine and chemicals. Herbal plants are becoming the top-selling phytotherapeutics globally. A plethora of preclinical studies have shown that hawthorn extracts possess cardioprotective and anti-atherosclerotic properties and contain major bioactive components identified as flavonoids, polyphenols, and oligomeric procyanidins. The underlying mechanisms are associated with reduced serum lipid contents, which involve inhibition of lipid absorption in intestines and cholesterol *de novo* synthesis in the liver, as well as promoting cholesterol efflux, which subsequentially suppresses lipid retention and plaque formation. Reduced lipid retention then results in a lower number of foam cells, which is the major source of reactive oxygen species (ROS) and inflammatory cytokines. This also helps to maintain the normal function of endothelial cells, including its permeability, which in turn halts the lipid and circulating macrophage/monocyte infiltration.

These cardioprotective actions of the hawthorn target various pathological conditions associated with atherosclerosis, which may offer great potential for synergistic effects. In addition to these experimental data, a great deal of knowledge has been acquired regarding the benefits of hawthorn on other CVDs, including heart failure and primary hypertension (Wang et al., 2013; Holubarsch et al., 2018). However, little known is about its effects on coronary atherosclerosis in the clinical arena.

The current guidelines for the treatment of acute coronary syndrome and secondary prevention recommend medications such as inhibitor of platelet activation and aggregation, lipid modification drugs, β-blockers, and inhibitors of the renin-angiotensin-aldosterone system (RAAS) (Amsterdam et al., 2014; Roffi et al., 2016; Chinese Medical Association Cardiovascular Branch & Editorial Board of Chinese Journal of Cardiovascular Diseases, 2017). The biological effects of hawthorns may serve the needs of these strategies. The pathophysiology of atherosclerosis is complex; therefore, a therapeutic approach capable of targeting multiple arms of the cardioprotective aspects would likely elicit greatest efficacy in reducing the morbidity and mortality associated with atherosclerosis. However, the lack of investigation on its synergistic effects, and explanations on the specific underlying mechanisms, raises controversies with respect to its therapeutic efficacy. This calls for further clarification on whether the mixture of hawthorn extracts may exert synergistic benefits compared to the pure compound or rather exacerbate side effects.

It is worth noting that extracts from hawthorn leaves have been recorded in the European pharmacopoeia (European Pharmacopoeia, 2017) and in the pharmacopoeia of the People’s Republic of China (Chinese Pharmacopoeia Commission, 2015), such as WS^®^ 1442 which has been extensively investigated in both preclinical and clinical studies. However by now, none of its effects on patients with coronary heart disease was reported. In China, several hawthorn leaf products for the treatment of angina and coronary heart disease have been approved by the National Medical Products Administration for sale (http://www.nmpa.gov.cn/WS04/CL2042/). Nevertheless, clinical and pharmacological data of these drugs are not enough to evaluate its efficacy at the present moment.

Furthermore, the current consensus on the primary and secondary prevention of coronary heart disease requires long-term use of various medications interacting with multiple targets. The combined application of the hawthorn with other medications may give rise to unexpected effects. Hence, high-quality trials with long-term follow-up are warranted to provide more compelling evidence of its safety and efficacy.

Taken together, the cardiovascular activities and clinical implications of hawthorn extracts have been extensively studied. Current evidence shows its potential as a phytotherapeutic agent adjuvant in conventional management of coronary heart disease. This requires further efforts to explore its clinical efficacy and safety for the treatment of atherosclerosis.

## Author Contributions

MW and LL contributed equally to this manuscript. YC, HL, MW, LL, SY, and YX participated in the conception of the review. YC and HL drafted the initial full manuscript. MW and LL edited the manuscript.

## Funding

This work was funded by the Beijing Natural Science Foundation (No.7172185), the National Key R&D Program of China (2018YFC1704901), and the National Natural Science Foundation of China (No. 81202805, 81973689, and 81573821).

## Conflict of Interest

The authors declare that the research was conducted in the absence of any commercial or financial relationships that could be construed as a potential conflict of interest.
